# A Personalized Home-Based Rehabilitation Program Using Exergames Combined With a Telerehabilitation App in a Chronic Stroke Survivor: Mixed Methods Case Study

**DOI:** 10.2196/26153

**Published:** 2021-08-31

**Authors:** Dorra Rakia Allegue, Dahlia Kairy, Johanne Higgins, Philippe S Archambault, Francois Michaud, William C Miller, Shane N Sweet, Michel Tousignant

**Affiliations:** 1 School of Rehabilitation Université de Montréal Montreal, QC Canada; 2 The Centre for Interdisciplinary Research in Rehabilitation of Greater Montreal Institut universitaire sur la réadaptation en déficience physique de Montréal Montreal, QC Canada; 3 Mission Universitaire de Tunisie Montreal, QC Canada; 4 School of Physical & Occupational Therapy McGill University Montreal, QC Canada; 5 Department of Electrical Engineering and Computer Engineering Université de Sherbrooke Sherbrooke, QC Canada; 6 Department of Occupational Science & Occupational Therapy University of British Columbia Vancouver, BC Canada; 7 Department of Kinesiology and Physical Education McGill University Montreal, QC Canada; 8 Faculty of Medicine and Health Sciences, School of Rehabilitation Université de Sherbrooke Sherbrooke, QC Canada; 9 Center of research on Aging Sherbrooke, QC Canada

**Keywords:** stroke, rehabilitation, virtual reality, video games, telerehabilitation, upper extremity, motivation

## Abstract

**Background:**

In Canada, only 11% of stroke survivors have access to outpatient and community-based rehabilitation after discharge from inpatient rehabilitation. Hence, innovative community-based strategies are needed to provide adequate postrehabilitation services. The VirTele program, which combines virtual reality exergames and a telerehabilitation app, was developed to provide stroke survivors with residual upper extremity deficits, the opportunity to participate in a personalized home rehabilitation program.

**Objective:**

This study aims to determine the feasibility of VirTele for remote upper extremity rehabilitation in a chronic stroke survivor; explore the preliminary efficacy of VirTele on upper extremity motor function, the amount and quality of upper extremity use, and impact on quality of life and motivation; and explore the determinants of behavioral intention and use behavior of VirTele along with indicators of empowerment.

**Methods:**

A 63-year-old male stroke survivor (3 years) with moderate upper extremity impairment participated in a 2-month VirTele intervention. He was instructed to use exergames (5 games for upper extremity) for 30 minutes, 5 times per week, and conduct videoconference sessions with a clinician at least once per week. Motivational interviewing was incorporated into VirTele to empower the participant to continue exercising and use his upper extremities in everyday activities. Upper extremity motor function (Fugl-Meyer Assessment–upper extremity), amount and quality of upper extremity use (Motor Activity Log-30), and impact on quality of life (Stroke Impact Scale-16) and motivation (Treatment Self-Regulation Questionnaire-15) were measured before (T1), after (T2) VirTele intervention, and during a 1- (T3) and 2-month (T4) follow-up period. Qualitative data were collected through logs and semistructured interviews. Feasibility data (eg, number and duration of videoconference sessions and adherence) were documented at the end of each week.

**Results:**

The participant completed 48 exergame sessions (33 hours) and 8 videoconference sessions. Results suggest that the VirTele intervention and the study protocol could be feasible for stroke survivors. The participant exhibited clinically meaningful improvements at T2 on the Fugl-Meyer and Stroke Impact Scale-16 and maintained these gains at T3 and T4. During the follow-up periods, the amount and quality of upper extremity use showed meaningful changes, suggesting more involvement of the affected upper extremity in daily activities. The participant demonstrated a high level of autonomous motivation, which may explain his adherence. Performance, effort, and social influence have meaningful weights in the behavioral intention of using VirTele. However, the lack of control of technical and organizational infrastructures may influence the long-term use of technology. At the end of the intervention, the participant demonstrated considerable empowerment at both the behavioral and capacity levels.

**Conclusions:**

VirTele was shown to be feasible for use in chronic stroke survivors for remote upper extremity rehabilitation. Meaningful determinants of behavioral intention and use behavior of VirTele were identified, and preliminary efficacy results are promising.

**International Registered Report Identifier (IRRID):**

RR2-10.2196/14629

## Introduction

### Background

Stroke can cause chronic sequelae in the hemiparetic upper extremity, requiring long-term rehabilitation care [1]. In Canada, only 11% of stroke survivors have access to outpatient and community-based rehabilitation after discharge from inpatient rehabilitation [2]. Stroke rehabilitation can help minimize impairments and promote independence during both the acute and chronic phases of stroke [3,4]. Hence, innovative community-based strategies are needed to provide adequate postrehabilitation services to stroke survivors in the community when these resources are not available. Consequently, virtual reality (VR) exergames are a promising alternative to traditional rehabilitation services, allowing continuous access to challenging exercises [5]. When combined with a telerehabilitation app [6], a follow-up with a clinician may be provided to adjust the difficulty level of the exergames and choice of exercises, according to the stroke survivor’s abilities and goals. The VirTele program—a program that combines VR exergames and a telerehabilitation app—was developed to provide stroke survivors with residual upper extremity deficits the opportunity to participate in a personalized rehabilitation program.

Motor and functional gains can be achieved during the chronic phase of stroke. However, it remains difficult to maintain these gains throughout time as they are essentially dependent on adherence to exercise guidelines and the availability of community-based resources, such as access to rehabilitation services, programs, and clinicians with stroke recovery expertise [4]. Therefore, rehabilitation programs developed for chronic stroke survivors should include strategies to encourage and motivate the use of the affected upper extremity through self-directed exercises and everyday activities [4,5]. Behavior change techniques (BCTs) were therefore incorporated into the VirTele program to empower users to continue exercising and using their upper extremities in everyday activities (eg, brushing their hair, getting dressed, and eating) [7]. Furthermore, there is increasing evidence that having a strong theoretical basis and using BCTs improves the efficacy of programs aimed at changing behaviors [7-9].

In light of current knowledge, the self-determination theory [10] was used to guide the clinician-stroke survivor interaction during videoconference sessions in the VirTele program. This theory stipulates that people tend to change their behavior when three of their needs are respected, including autonomy (the ability to organize their behavior according to their own aspirations and values), connectivity (the sense of belonging and the desire to be supported), and competencies (belief in their skills and abilities) [10]. Thus, the clinician was trained to consider these three psychological needs when interacting with a stroke survivor. In addition, BCTs (eg, 1.1. Goal setting, 1.4. Action planning, and 1.5. Review behavior goals) [7] and motivational techniques (open-ended questions, affirmation, reflective listening, and summary statements) [11] may be used to enhance stroke survivors’ motivation and adherence to VirTele exercises [12,13].

As we intend to implement the VirTele program in stroke survivors’ communities, factors that could influence behavioral intention and use behavior regarding the intervention must be explored. This study was conducted in the context of a larger study before starting a randomized clinical trial to inform the development of evaluation and intervention protocols [14]. The clinical trial was registered at ClinicalTrials.gov (NCT03759106).

### Objectives

The objectives of this study are (1) to determine the feasibility of using a telerehabilitation app combined with an exergame for a remote upper extremity rehabilitation program in a chronic stroke survivor; (2) explore the preliminary efficacy of VirTele on upper extremity motor function, the amount and quality of upper extremity use, and impact on quality of life and motivation; and (3) explore the determinants of behavioral intention and use behavior of VirTele along with indicators of empowerment.

## Methods

### Overview

A case study using a mixed methods design was conducted before the start of a randomized clinical trial. A quantitative approach was used to collect feasibility and preliminary efficacy data before and after the VirTele intervention and during a 1- and 2-month follow-up period. A qualitative approach was used to explore the factors that influenced the use of the technology and its impact on the participants’ empowerment. This study was approved by the research ethics boards of the Center for Interdisciplinary Research in Rehabilitation of Greater Montreal (June 28, 2018).

### Participant

A 63-year-old male stroke survivor (3 years) from the United States participated in this study. His upper extremity score on the Chedoke McMaster Stroke Assessment Scale indicated moderate impairment (arm stage 6). The participant was no longer receiving rehabilitation services and had an active lifestyle (swimming, walking, and fitness training). He was very comfortable with computers as he had a professional information technology background and used a computer one or more times a week.

After reading about our registered protocol at ClinicalTrials.gov, he approached our research team via email. At that time, the development and testing phases of the rehabilitation program and technology with VirTele were still ongoing. The participant was enthusiastic about playing an expert role in the study and testing the VirTele remote rehabilitation program in a setting similar to that of the proposed research study. He was eager to help identify the best way to combine telerehabilitation and videogames for home use to optimize upper extremity functional and motor recovery following a stroke. In doing so, he would assist in developing a final VirTele rehabilitation training that could be used as an intervention model for our future randomized clinical trial. Given the remote nature of the VirTele program, we were able to conduct the study with the participant remotely. The participant agreed to contribute to preliminary data collection and will not be included in future clinical trials. He provided informed consent before starting the case study, as per the Centre for Interdisciplinary Research in Rehabilitation of Greater Montreal’s institutional ethics review board.

### VirTele Intervention Protocol

#### Overview

The participant was invited to complete a home-based, 2-month rehabilitation training program using a telerehabilitation app combined with a nonimmersive VR exergame. The equipment necessary to operate the technology properly included internet access, a computer (desktop or laptop), a video camera, and a Kinect camera. The participant already owned all the equipment except for the Kinect camera, which was sent to him. Before starting the intervention, the participant was trained on using the telerehabilitation app Reacts (Innovative Imaging Technologies and Reacts) [15] and the VR exergame Jintronix (Jintronix Inc) [16], two systems previously used by the research team.

The program included 30-minute exergame sessions 5 times per week for 2 months (approximately 20 hours) and 1-hour videoconference sessions with a clinician one to three times per week for 2 months. However, it was up to the clinician to determine the frequency of follow-up. The clinician based that decision on the stroke survivor’s behavior (adherence to the exergames and use of the affected arm in daily life activities) and needs (support for exergames use and discomfort). This approach intends to maintain the participant’s motivation, ensure that the exergames are adequately tailored, monitor functional use of the upper extremity, and develop strategies to increase the participant’s autonomy in an effort to maintain the participant’s activity level after the study ended. The clinician (DRA; physiotherapist) is part of the research team and has 4 years of experience working with a stroke population in research settings.

#### Exergame Sessions

We used the Jintronix-VR exergame (Jintronix Inc), which included 5 types of upper extremity games at the time (*Space Race*, *Fish Frenzy*, *Pop Clap*, *Catch and Carry an apple*, and *Kitchen clean up*) [16]. The practice involved reaching virtual target objects and moving them following a specific trajectory preselected by the clinician based on the participant’s level of impairment. For example, with the *Fish Frenzy* task, the participant must successfully move a fish along a designated path by controlling the position of the affected hand; in the *Kitchen clean up* task, the participant must pick up various pieces of cutlery and dishes and put them away in the appropriate drawer or shelf. Although performing the virtual tasks, the participant can see his own virtual hand displayed on the screen in games such as *Pop Clap*, *Catch and Carry an apple*, and *Kitchen clean up*. In other games (*Space Race* and *Fish Frenzy*), the hand is replaced by a virtual object (fish or spaceship). The exergame platform also provides visual feedback on the game score, the remaining time to succeed in the task and the warning signal for each unsuccessful attempt, as well as auditory feedback reflecting successful or unsuccessful outcomes of the accomplished movement. The exergame sessions follow a structured training protocol with tasks of increasing difficulty using various parameters, such as the number of rounds, repetitions, time, speed, precision, and shape of objects, adjusted weekly by the clinician during videoconference meetings. Overall, the exergames were chosen based on the participant’s preferences and progress.

#### Gameplay Overview

**Figure 1:**
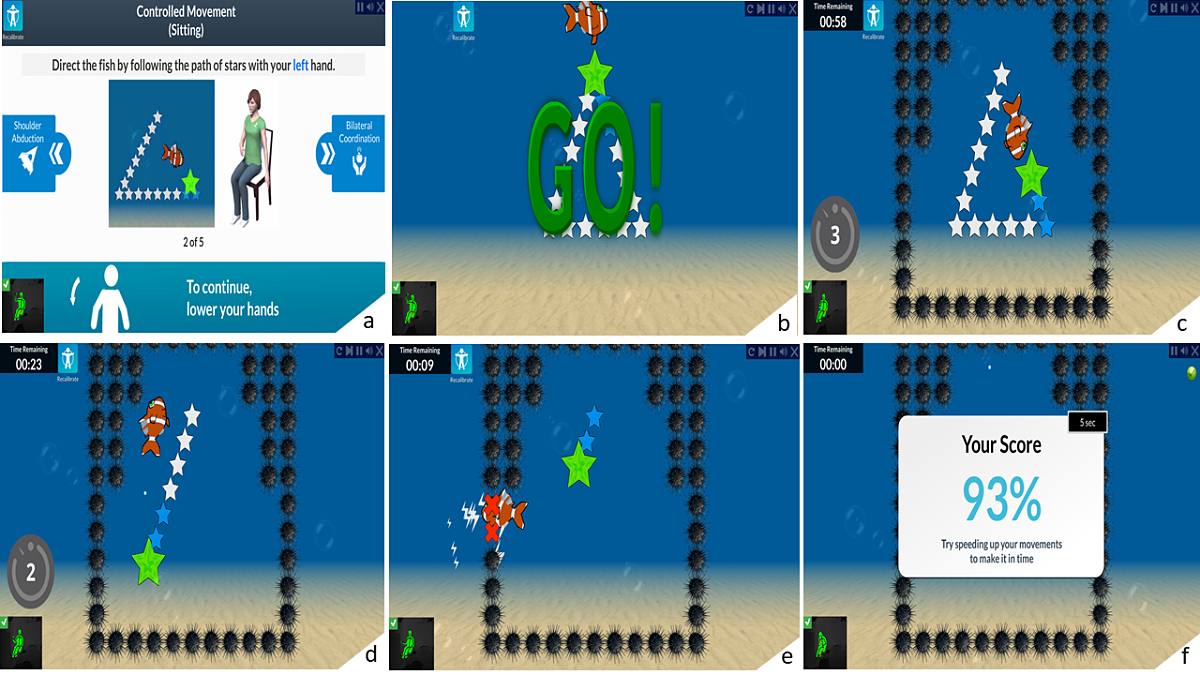
All phases of the Fish Frenzy task are depicted from (a) to (f). At the start of the game session, an example of the gameplay with a human model is shown on the screen. At the GO signal, the stroke survivor starts moving the fish to follow a path or catch the stars. The stroke survivor must complete the journey as fast as he can and repeat the same journey as often as possible for a predetermined period, as assigned by the clinician. The stroke survivor has to control his upper extremity when moving the fish to avoid touching the black objects surrounding the stars, which results in a loss of points. When finished, the final score is shown.

The game starts on a menu screen in which a predetermined program training session can be launched. After clicking start, the Kinect camera starts calibrating the initial stroke survivor’s position, capturing the user skeleton with markerless motion sensors [17]. An example of the gameplay overview of the Fish Frenzy task is shown in Figure 1. Screenshots of the gameplay of the other games are provided in Multimedia Appendix 1.

#### Telerehabilitation

**Figure 2:**
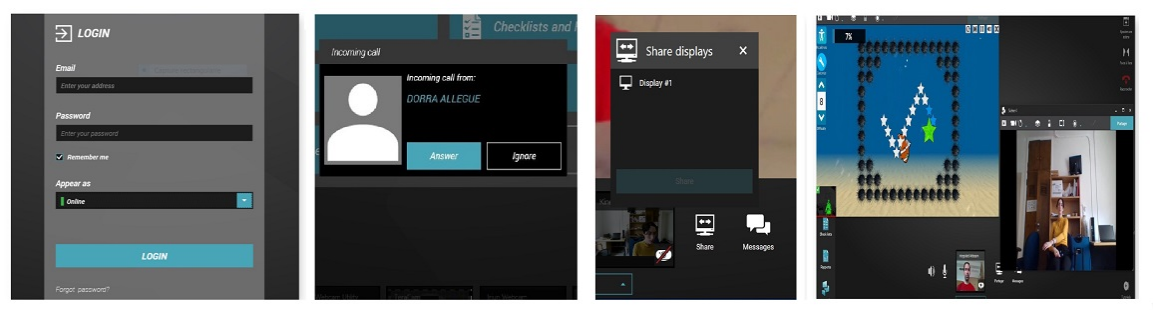
All phases of running the exergames and the videoconference system simultaneously are depicted from left to right. After entering their email and password into Reacts, the stroke survivor waits for the clinician’s call and then clicks on the answer button to start the videoconference session. Once the videoconference begins, they must click on the share button. After launching screen sharing, the stroke survivors may start playing the games. In this phase, the clinician can see the gameplay overview and the stroke survivor.

For the telerehabilitation app, we used Reacts (Innovative Imaging Technologies Inc and Reacts) [15], which allows videoconferencing and secure sharing of participants’ health information [15]. The telerehabilitation app was used for remote supervision, monitoring, and rehabilitation purposes. An algorithm design was used to run the telerehabilitation app and exergame simultaneously, allowing the clinician to see the participant performing the exercises and view the game screen simultaneously, making the VirTele program unique. The videoconference session allows the clinician to provide the participant important instructions and advice during the games, for example, to correct their posture or their upper extremity position and observe the quality of the participant’s upper extremity movements. In studies using VR systems for home-based rehabilitation [18-24], supervision is not always provided [16,18], and when it is offered, it is delivered by telephone [18,19,23] and rarely synchronized with the time when the participant is performing the games [21,24]. All phases of running the exergames and the videoconference system are shown in Figure 2.

### Motivational Interviewing

Motor and functional gains can be achieved during the chronic stroke phase; however, for long-term maintenance, rehabilitation programs should include strategies to encourage and motivate the use of the affected upper extremities through self-directed exercises and everyday activities [4,5]. Therefore, motivational interviewing based on BCTs was incorporated into the videoconference sessions to ensure that shared decision-making and empowerment were consistently integrated into the VirTele intervention. The BCTs included were as follows: 1.1. Goal setting, 1.4. Action planning, 1.5. Review behavior goals, 4.1. Instruction on how to perform the behavior, 8.1. Behavioral practice, 6.1. Demonstration of the behavior, 2.2. Feedback on behavior, 2.7. Feedback on outcomes of behavior, 1.6. Discrepancy between current behavior and goal, 1.2. Problem solving, 2.3. Self-monitoring of behavior, 9.2. Pros and cons, 5.1. Information about health consequences, and 15.1 Verbal persuasion about capability [7]. Motivational interviewing techniques included open-ended questions, affirmation, reflective listening, and summary statements [11].

In addition to exergames, the program included supplementary daily activities to meet participants’ individual goals, such as hand dexterity (eg, grasping, holding, and pinching) using the resources available within the participant’s home.

Thus, many tools available through the telerehabilitation app (document sharing) and the exergame (computerized participant logs such as active time, compensation, and scores during each game) were used by the clinician for encouragement, goal setting, tracking of upper extremity use in daily activities, monitoring of exergame adherence (duration, intensity, and type), and tailoring the exergames to the participant’s abilities.

In accordance with the self-determination theory [10], the clinician was trained to consider the three psychological needs when interacting with the stroke survivor by including him in every step of the decision-making process related to the treatment plan, respecting his autonomy, and valuing his skills and ability to achieve his goals.

The clinician received training in motivational interviewing before starting the intervention and used standardized interview guidelines during each videoconference session. The motivational interview guidelines were standardized to facilitate their use in further research and better monitor the use of BCTs. In that sense, predefined questions were prepared and applied concerning the stroke survivor’s three psychological needs (autonomy, competence, and connectivity). The choice of questions varied depending on the stroke survivor’s behavior state (total adherence or nonadherence to VirTele). The questions were provided as examples, but the clinician was free to reformulate them. Further details can be found in Multimedia Appendix 2.

### Data Collection

#### Functional Performance and Self-reported Data

For this case study, progress was assessed remotely using the videoconference system Reacts (Innovative Imaging Technologies Inc and Reacts) at baseline, at the end of the 2-month intervention, and after a 1- and 2-month follow-up period. Performance-based measures included the Fugl-Meyer Assessment–Upper Extremity (FMA-UE) [25,26] motor function score, which captures synergy, coordination or speed, and the sensorimotor function of the upper extremity, wrist, and hand. The FMA-UE motor function score has been shown to be valid in a stroke population [27] and reliable for remote evaluation (video observation of the scale administration by an evaluator) [28]. Given the participant’s distance, we tested the feasibility of administering the FMA-UE (motor function) remotely, without the presence of an evaluator on site with the participant. For that purpose, the score was adjusted to 60 as the reflex activity component was removed from the scale (which was retained in other studies, including on-site evaluations) [28,29].

Self-reported questionnaires included the Motor Activity Log (MAL)-30 [30,31], the Stroke Impact Scale (SIS)-16 [32,33], and the Treatment Self-Regulation Questionnaire (TSRQ)-15 [34]. The MAL is a self-reported measure that rates the quality (MAL–quality of use subscale) and frequency of use (MAL–amount of use subscale) of the affected upper extremity in 30 everyday functional tasks [30,31]. The MAL includes several versions (MAL-14, MAL-26, MAL-28, and MAL-12), which vary according to the number of items, but the original version of the MAL-30 is the most encompassing in terms of tasks and best meets our study objectives [35]. The MAL-30 demonstrated high reliability and validity in a poststroke population [36]. The impact on quality of life was determined using the SIS, a stroke-specific, self-reported health status measure consisting of 16 items. The SIS evaluates the impact of stroke on 3 dimensions: *hand function*, *activities of daily living*, and *mobility* [32,33]. The SIS-16 has been shown to have good reliability and validity [37]. Motivation was measured using the TSRQ, a 15-item questionnaire developed to measure treatment motivation, consistent with the self-determination theory [10]. The TSRQ captures different processes of motivation through 4 subscales: *autonomous motivation*, *amotivation*, *external regulation*, and *introjected regulation*. First, external regulation is the lowest level of the regulatory process that contributes to short-term changes [38]. During this process, the person behaves in such a way that they obtain a reward or avoid punishment; this choice is controlled by certain consequences, and they do not act according to their own wishes [38]. Second, introjected regulation describes a controlled motivation regulated by internal pressures, where a person behaves to avoid feeling guilty or proud [38]. Ultimately, autonomous motivation corresponds to the state of internalization of extrinsic motivation, where the person is in full agreement with the new changes, and their self-regulated behavior becomes autonomous [38]. Finally, amotivation describes a lack of motivation to pursue these changes.

A previous version of the TSRQ, a 13-item questionnaire, did not include the amotivation subscale, which is an important aspect to capture in our study, thus explaining our choice of the 15-item version. The TSRQ-15 has been shown to be reliable and valid across health care contexts [34], including rehabilitation [39].

The participant received the questionnaires through the telerehabilitation platform, which allows secure document sharing. Once the questionnaires were filled out, we met the participant via a videoconference to check his answers (to ensure that the questions were well understood) and ask for clarifications if necessary. This step was only taken in this case study, as we assessed the feasibility of the evaluation process, including remote questionnaire administration.

Sociodemographic variables were also collected at baseline: age, sex, civil status, years of education, primary occupation, date of stroke, time since stroke, side of stroke, and handedness. We also documented all physical and leisure activities (types and duration) accomplished before, during, and after the intervention.

#### Qualitative Data

Qualitative data were collected throughout the study using different sources (logs, videos, and email correspondence). A semidirected interview was conducted at the end of the intervention to identify the factors that influenced the behavioral intention and use behavior of VirTele and capture the participant empowerment indicators. The interview guide was developed based on the principles of the self-determination theory [10] and the Unified Theory of Acceptance and Use of Technology (UTAUT) [40]. The UTAUT highlights three direct determinants of behavioral intention (social influence, performance expectancy, effort expectancy, and facilitating conditions) and two direct determinants of use behavior (facilitating conditions and behavioral intention) of a new technology [40]. In addition, 4 moderators were described in this theory, including age, gender, experience, and voluntariness of use [40]. The UTAUT has been validated and consistently explains use behavior in various settings (research and technology) [41].

The constructs of the UTAUT [40] were used to inform the determinants that influenced the behavioral intention and use behavior of VirTele. Operational definitions were used for each theoretical construct to capture the context in which VirTele was used and better comprehend the environment-related factors. The operational definitions of the constructs are as follows: *performance* is defined as the degree to which a participant believes that VirTele would enhance or has enhanced their exercise performance (eg, duration, frequency, difficulty, goals, and expected outcomes) to optimize rehabilitation of the upper extremity. This definition embodies both expected and actual performance, as it aims to capture the participant’s expectancy of VirTele regarding exercise performance and the actual exercise performance after using VirTele, which could impact their motivation to continue using the system. *Effort* is defined as the degree of ease or complexity related to VirTele use and encompasses both expected and experienced efforts. *Social influence* is defined as the degree to which an individual entourage agreement or disagreement may affect their use of VirTele. *Facilitating conditions and obstacles* are defined as the factors that may facilitate or inhibit the use of VR-telerehabilitation technology, including control of internal constraints (capacity and knowledge to use new technology) and external constraints (technical and organizational infrastructure). The self-determination theory constructs were used to inform the participant empowerment during the VirTele program.

#### Feasibility Data

Descriptive data relating to the VirTele rehabilitation training were documented in a standardized form at the end of each week. These data included the number and duration of videoconference sessions, adherence to the exergames (duration and frequency), technical difficulties, and adverse events (pain, falls, motion sickness, dizziness, exertion, fatigue, and headaches). These data were collected from logs completed by the clinician and from the exergame and telerehabilitation portals.

### Data Analysis

Descriptive analyses were conducted for performance-based measures and self-reported data. The interview was audio-recorded, transcribed verbatim, and analyzed through the lens of the self-determination theory [10] and the UTAUT [40]. Content analysis was applied to identify the factors that influenced behavioral intention and use behavior of VirTele and significant indicators of empowerment that could result from the use of the VirTele program, including motivational strategies. The identified empowerment indicators were categorized into capacities and behaviors based on the conceptual model of patient empowerment [42]. When relevant, themes identified through the qualitative analysis were used to deepen and explain the quantitative findings.

For the scientific rigor of qualitative data, the principles of Lincoln and Guba [43] were applied. Audit trail and verification were performed to ensure confirmability. External verification by team members was applied to ensure credibility. Reliability was achieved through verification by 3 coders of a part of the data. For transferability, reflexive notes and a detailed description of the context of the intervention were provided.

## Results

### Functional Performance and Self-reported Data

The FMA-UE (motor function component) exhibited a significant increase in the total score from baseline (T1) to postintervention (T2; total score difference 6; Table 1), which is within the estimated minimal clinically important difference (MCID) range of 4.25 to 7.25, specifically for the upper extremity motor function component [44]. The FMA-UE total score was maintained during the follow-up period at T3 and T4 and may suggest a slight increase, although there were no significant changes beyond the postintervention results at T2 (Table 1).

**Table 1:** Functional performance and self-reported measures.

Outcome measures			T1		T2		T3		T4		Score direction
Fugl-Meyer Assessment–Upper Extremity motor function (score from 0 to 60)			49		55		56		57		High score: better motricity of the affected upper extremity
**Motor Activity Log (score from 0 to 5)**											
	Amount of use	1.53		2.45		2.72		3.26		High score: higher frequency of use of the affected upper extremity	
	Quality of use	1.43		2.29		2.65		2.95		High score: better quality of movement of the affected upper extremity	
**Stroke Impact Scale (score from 0 to 100)**											
	Hand function	0		25		25		50		Low score: high impact on quality of life	
	Mobility	75		71.43		64.28		64.28		Low score: high impact on quality of life	
	Activities of daily living	68.75		71.87		87.5		84.37		Low score: high impact on quality of life	
**Treatment Self-Regulation Questionnaire**											
	Autonomous motivation (score from 0 to 42)	42		42		42		42		High score: high level of autonomous motivation	
	Introjected regulation (score from 0 to 14)	8		8		2		5		High score: high level of introjected motivation	
	External regulation (score from 0 to 28)	6		10		4		8		High score: high level of external motivation	
	Amotivation (score from 0 to 21)	3		3		3		4		High score: high level of amotivation	

The total score on the MAL–amount of use increased to 2.45 at T2, close to the score of 2.5 (Table 1), predicting 50% or greater perception of recovery in the affected upper extremity (based on a scale of 0% to 100% of SIS–section perceived recovery) [45]. During the follow-up period, improvement in the amount of use scale was maintained and exceeded the score of 2.5 at T3 and T4. The change in the MAL–quality of use at T2 (total score difference 0.86) was not clinically relevant (MCID is between 1.0 and 1.1 [31]) but was significantly different from baseline at T3 (total score difference 1.22) and T4 (total score difference 1.52; Table 1).

The SIS revealed improvements in hand function and activities of daily living dimensions with a meaningful difference from baseline to T4 (Table 1), exceeding both the MCID range of 9.4 to 14.1 [33]. There was no improvement in the SIS mobility dimension, with a decreasing tendency over time (Table 1).

The TSRQ showed a high score on the autonomous motivation subscale (score=42/42), which was maintained during the follow-up period at T3 and T4. The amotivation subscale showed a low score (score=3/21) from baseline to the follow-up period at T3 and increased by 1 point at T4 (score=4/21). The external regulation subscale showed an increase in score from baseline (T1) to postintervention (T2; score from 6 to 10/28), followed by a decrease at T3 (score=4/28) and an increasing tendency at T4 (score=8/28). The introjected regulation subscale showed a moderate score (score=8/14), which was maintained from baseline to postintervention T2 and decreased during the follow-up period.

### Qualitative Data

#### Determinants of Behavioral Intention and Use Behavior of VirTele

Consistent with the UTAUT, we identified four major determinants from the directive content analysis, namely, performance, effort, social influence, facilitating conditions, and obstacles.

##### Performance

Regarding performance, the participant raised many relative advantages of VirTele compared with standard therapy (exchange of messages, videoconference, follow-up by the clinician, no limited time of therapy, time to reflect, correct, and discuss with the clinician) and perceived an improvement in arm functional performance and impact on daily activities, including making his bed, putting socks on, doing laundry, and shopping.

##### Effort

As for effort, the participant was very comfortable using VirTele and reported only minor issues related to the audio and video being cut off, which he resolved himself. Effort was highly moderated by participants’ previous experiences with information technology. In fact, the participant’s background in information technology facilitated his VirTele use and management of the issues he encountered.

##### Social Influence

Concerning social influence, it was important for the participant to share his VirTele experience with his friends and family, and receiving positive feedback encouraged him to continue using the technology.

##### Facilitating Conditions and Obstacles

The qualitative results suggest that the participant believed that he had the necessary knowledge to use VirTele but raised potential factors that could influence the long-term use of the technology: the lack of control of technical infrastructures (internet access, quality of internet service [high speed], and the method to modify the exergames) and organizational infrastructure (access difficulty to VR at the individual level and limited health system resources). In addition, the participant reported a lack of compatibility of VirTele with needs in terms of game design (choice of avatar and background color) and previous experience (interoperability).

In addition to the pre-established determinants of the UTAUT, three new constructs emerged, including attitudes toward VirTele (satisfaction with the telerehabilitation platform, satisfaction with the exercises provided during the intervention, and satisfaction with the VR exergames), attitudes toward the affected upper extremity (anxiety related to upper extremity improvement and exercising), and attitudes toward information technology (eager to experiment with VR).

**Figure 3:**
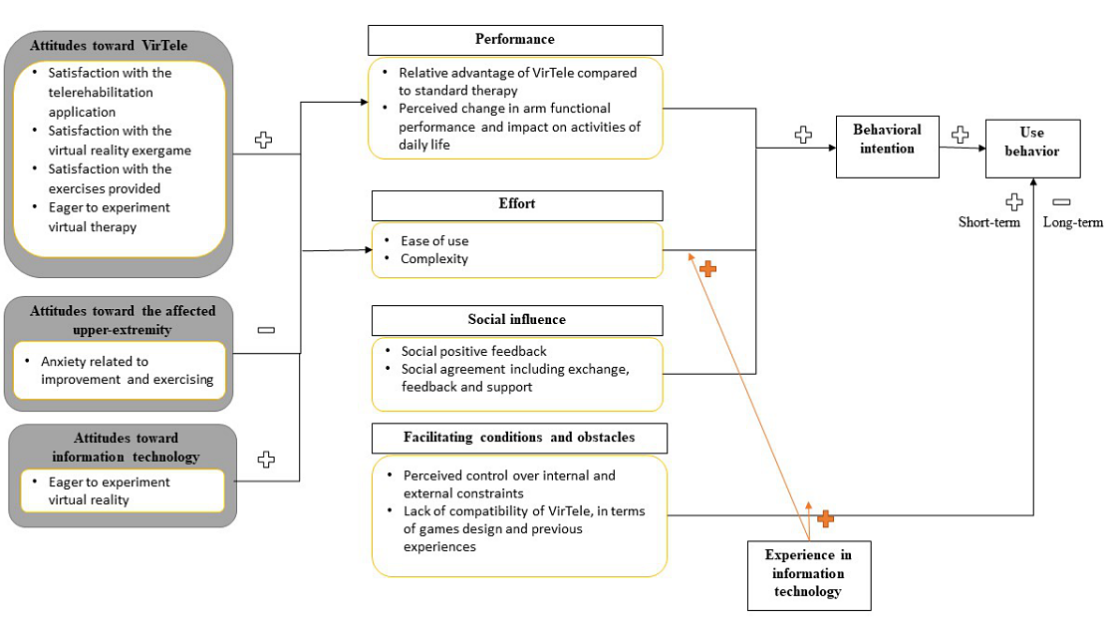
Factors that influence behavioral intention and use behavior of VirTele.

A model of how these four determinants and attitudinal factors interact with behavioral intention and use behavior is presented in Figure 3.

#### Empowerment

Of the eight videoconference sessions, two sessions focused on motivational interviewing. The first one was conducted after the participant reported discomfort to find a strategy to overcome the problem and motivate him to gradually resume the exercises. The second one was conducted 2 weeks later to maintain his adherence to the exercises using different motivational techniques such as goal planning, exploration of ambivalence, valuation, and reflective listening.

In the other six sessions, the clinician usually started the videoconference with remote supervision of the participant’s performance, adjustment of the difficulty level of the preselected games, and finally ended with motivational interviewing. During the videoconference sessions, the participant took an active role and participated in every decision related to his rehabilitation program, including the choice of games, difficulty level, and strategies to improve daily life activities.

Relevant empowerment indicators were identified and categorized into capacities and behaviors. Detailed descriptions of these indicators are provided in Textbox 1.

**Description of empowerment indicators in terms of capacities and behaviors.**

**Description of indicators in terms of capacities and behaviors**

- Attitudes
  High expectation of improvement
  Motivation to continue exercising despite the difficulties
  Willingness to communicate and exchanges ideas of exercises modalities
  Willingness to participate in patient support or advocacy groups
- Self-awareness
  Importance of exercise
  Continue exercising despite difficulties
  Awareness that recovery may require long and hard work
- Perceived personal control of health and health care
  Limited access to health care services
  Frustration because of lack of access to health services
- Sense of meaning and coherence about health condition
  No complaints about the functional stage attained and gratefulness
- Health literacy
  Comprehension of the neuroplasticity phenomenon and its role in stroke recovery
- Self-management of own health care
  Using a keyboard instead of a touching screen to exercise fingers
  Management of time between autonomous game sessions, supervised sessions with a clinician, and fitness program
- Participation in decision-making
  Discussion of the exercise’s parameters with the clinician
  Discussion of the difficulties encountered and related solutions
- Self-empowerment
  Use the internet to collect information and support from other survivors and get other perspectives
  Use the internet to collect information about other virtual reality-based rehabilitation and contact clinician
  Participation in a course given at the rehabilitation school at Université de Montreal (Montreal, Canada) as a speaker to share his VirTele experience with students and clinicians

### Feasibility Data

#### Adherence to the VirTele Program

During the 2-month intervention, the participant completed 48 sessions of 33 hours and 15 minutes of active games and 8 videoconference sessions with a clinician. As the participant was very comfortable with the technology and used the exergames more than expected, the clinician did not feel the need to meet with him more than once a week. During the fourth week, no meeting occurred, and the meeting was rescheduled during the fifth week (which included 2 meetings).

The 48 sessions included 6 supervised game sessions with a clinician and 42 autonomous game sessions. The time spent on each game and the number of repetitions are listed in Table 2. The participant was encouraged to play the games five times a week. However, he was free to repeat game sessions whenever he wanted.

**Table 2:** Time spent and the total number of repetitions achieved in the exergames.

Exergames	Active time playing the game (hours)	Total number of repetitions^a^
Fish Frenzy	4.48	2581
Catch and Carry an Apple	3.8	2418
Kitchen Clean Up	2.083	674
Pop Clap	11.116	10,304
Space Race	11.76	14,364
Total	33.25	30,341

^a^Reflects the number of successful tasks or movements completed during a game.

During the first month of VirTele, the participant was afraid of using his upper extremity in daily life activities (fear of dropping objects, difficulty in using upper extremity, or pain) and was only manipulating virtual objects during the exergames. Nonetheless, during the second month, the participant demonstrated more frequent use of the affected upper extremity to assist the healthy hand in carrying out a task, for example, drinking from a glass using the help of a finger of the affected hand and using the affected upper extremity to answer the phone (with a touch screen). The clinician encouraged and praised the participant in achieving these small steps to maintain his motivation to continue practicing, even after the end of VirTele.

#### Technical Challenges and Adverse Events

The participant quickly became familiar with the VirTele technology, including the use of the exergame and the telerehabilitation app. The noninteroperability of the exergame software with the participant’s computer (iMac, Apple) and his unfamiliarity with Windows operating systems did not prevent him from finding a solution to make the game work. In fact, the participant managed to divide his computer (iMac, Apple) hard drive into 2 parts to run Windows 10 and use the exergame.

Similarly, the participant encountered difficulties with the internet connection (video or sound cut off during the videoconference or poor connection) and log in process. However, he always managed to find a solution to each problem, for example, reset the modem or router, restart the apps, or reset the password, as he mentioned:

I didn’t have any problem in setting, as well as using and from time to time, when there is an update...when the system said: “wrong password”, I can reset the password and everything...remember we had a little bit of an issue with the audio, I was trying to put us in a video conference on my computer but it didn’t work and I tried and tried and finally I said fine I will use my iPad. It is a small screen but still on the iPad.

Overall, the participant appreciated exercising with the exergame and had suggestions to better enjoy the game experience. For example, he preferred seeing his own hand or an image of a realistic hand on the screen, instead of the glove or the spaceship, and he was more drawn to realistic scenes than fictional scenarios (coloring stars with a pen instead of catching stars with a fish).

No adverse events occurred during the first few weeks of intervention. However, the participant reported an increased feeling of heaviness in the affected upper extremity during the fourth week of the intervention. The clinician in charge suggested that the participant used stretching postures at the beginning and end of the games to prevent discomfort. These were demonstrated during the videoconference sessions and relieved discomfort.

## Discussion

### Principal Findings

We addressed three objectives in this case study: (1) determine the feasibility of using a telerehabilitation app combined with an exergame for a remote upper extremity rehabilitation program in a chronic stroke survivor; (2) explore the preliminary efficacy of VirTele on upper extremity motor function, amount and quality of use, impact on quality of life and motivation; and (3) explore the determinants of behavioral intention and use behavior of VirTele and indicators of participant empowerment.

### Adherence to VirTele, Technical Challenges, and Preliminary Efficacy Results

During the 2-month intervention, the participant demonstrated complete adherence to VirTele, completing 48 sessions or 33 hours and 15 minutes of active games, surpassing the minimum 20 hours planned for the program. A minimum of 15 hours is suggested for an intervention aiming at a moderate improvement of activities related to daily living following a stroke [46]. The 48 sessions included six supervised game sessions with a clinician and 42 autonomous game sessions. In addition to the exercises provided, the participant participated in eight videoconference sessions with a clinician for monitoring and empowerment purposes.

Although the participant encountered some technical challenges related to interoperability, internet connection issues (interruption of the video or sound, poor connection), and the login procedure (forgotten password), he was able to find a solution and use both the exergame and the telerehabilitation app throughout the 2 months. Furthermore, the participant suggested some improvements for the avatar and background, which he felt would render the game more meaningful. In addition, he was more drawn to realistic scenes and avatars, which is consistent with the findings of a previous study that indicated that players preferred simulated real-world games [47]. The sense of presence in a virtual environment or the degree to which an individual perceives the environment as real may affect the participant’s engagement and motivation [48,49]. Thus, it is important to consider certain elements, such as the background, avatar design, and the scene’s realism, when developing a VR system for rehabilitation use [48]. Fortunately, the lack of these elements did not decrease the participant’s compliance with or commitment to performing the exercises, given that he spent 11 hours using the Space Race game.

Regarding the combined use of a telerehabilitation app and an exergame, the results of this case study suggest that the VirTele intervention and study protocol could be feasible with stroke participants, although it is important to ensure that it will be accessible to participants who may be less motivated and technologically savvy. Moreover, the participant appreciated the follow-up by the clinician when playing the exergames and described this room for maneuver in VirTele as *the time to reflect*. In fact, while the clinician was supervising, the participant was able to identify his errors during the exercises (compensatory movements), discuss the solutions, adjust the difficulty level as needed, and correct his posture and upper extremity movements. This may have prevented him from reproducing a pathological pattern of movement when playing alone. The participant exhibited clinically meaningful improvements at T2 in the motor function dimension of the FMA-UE and the hand function dimension of the SIS. Not surprisingly, there was no improvement in the SIS mobility dimension, which addresses the impact of stroke on lower extremity mobility, further indicating the impact of the intervention on upper extremity motor function. The FMA-UE (motor function) and the SIS (hand function) scores were maintained at T3 and T4, followed by a relevant increase in the SIS score (daily living activities), suggesting maintenance of motor gains throughout time, contrary to the findings of a previous study using VR exergames without supervision and a motivational approach [6].

During the follow-up period, there was a meaningful change in the MAL–amount of use subscale, suggesting more involvement of the affected upper extremity in daily activities, along with a meaningful improvement in the SIS activities of daily living dimension at T3 and the SIS hand function dimension at T4. These results suggest that a transfer pattern occurred between the virtual and daily life activities, as the participant showed meaningful improvements in daily life activities and amount of use of the upper extremity following 2 consecutive months after completing the VirTele program.

Although we had predetermined that 20 hours of active games within the VirTele program should be performed, the participant decided to do more (33 hours and 15 minutes). A much higher number of repetitions may have positively affected the upper extremity motor function. In fact, a meta-analysis of 20 randomized clinical trials, including 2686 stroke patients (acute, postacute, and chronic), supports the presence of a dose-response relationship between intense practice (therapy time ranged from 132 minutes to 6186 minutes) and better outcomes [50]. However, it is important to note that all users may not use VirTele at this level of intensity. More research is required to determine the amount of time required to obtain optimal results.

The MAL–quality of use subscale showed no change in movement quality at the end of the program but exhibited a meaningful improvement during the follow-up period. In fact, during the 2-month training, the participant did not use his affected hand sufficiently during daily life activities (pain and fear) and was mostly manipulating virtual objects during the exergame, with no force or touch feedback, which may have limited the integration of somatosensory information that contributes to motor learning [5]. During the follow-up period, the participant stopped using the exergame and was more involved in daily life activities, more frequently using his upper extremity to manipulate real-life objects, which involves somatosensory feedback that may favorably affect the movement quality of the affected upper extremity [5]. These results highlight the necessity of transferring virtual activities to real-life activities to avoid a potential gap in somatosensory information affluence.

### Participant Motivation

The participant exhibited a lot of enthusiasm from the day he contacted our research team and demonstrated a high level of autonomous motivation (score=42/42) throughout the entire intervention, which may partly explain the high level of adherence to the VirTele rehabilitation program. The TSRQ showed a low level of external regulation, meaning that the participant made autonomous choices regarding his use of VirTele and completion of the exercises and was not influenced by certain consequences (“I want others to approve of me”) [5] or pressure (“I feel pressure from others to do so”) [34]. However, the external regulation score increased from 6 to 10 during the VirTele intervention. This could have possibly resulted from the desire to satisfy the research team or the clinician, leading to short-term changes in the participant’s behavior (eg, better adherence to VirTele and higher motivation), improving upper extremity motor scores. However, the long-term maintenance of motor gains involves continuous adherence to exercises (after the end of VirTele) and maintaining motivation despite the lack of external factors (eg, encouragement by the clinician).

Similarly, the TSRQ showed a medium level of introjected regulation (score=8/14), meaning that the participant partially introjected and self-identified with the values linked to the VirTele rehabilitation program. This suggests that the participant possibly integrated the potential benefits of the exercises with his affected upper extremity and identified aspects of the VirTele program that could match his own values and beliefs, potentially affecting the long-term behavioral use of the technology.

### Determinants of Behavioral Intention and Use Behavior of VirTele

Performance, effort, and social influence were meaningfully weighed in the behavioral intention use of VirTele. The relative advantages of the VirTele program compared with standard therapy and the perceived improvement in daily life activities facilitated the use performance of the VirTele program. The participant’s level of comfort with the technology positively influenced the effort determinant. The additional constructs of the UTAUT model were not identified as direct determinants of behavioral intention or use behavior. According to Venkatesh et al [51], attitudinal constructs may indirectly affect behavioral intention and use behavior through performance and effort constructs.

In fact, the participant’s attitude toward VirTele, the affected upper extremity, and information technology indirectly influenced behavioral intention through a positive and negative impact on effort and performance determinants. Owing to the participant’s experience in information technology, he may have been more sensitive to some aspects of the exergame design (eg, avatar and background) and the operating system within the technology, although these aspects did not affect his compliance with the VirTele program.

### Empowerment Indicators

The participant demonstrated considerable empowerment on the behavior and capacity levels, which may have positively affected his adherence to the VirTele rehabilitation program and explain the meaningful improvement in many clinical outcomes. The self-determination theory was used to inform the VirTele program by identifying the main constructs that target the desired behaviors (increase in adherence to the VirTele program and increased use of the affected upper extremity in daily life activities) and providing the means for developing component intervention techniques such as motivational interviewing. Health behavior change literature mentions numerous advantages of applying theory to interventions [52-54], including stronger effects [52], which may corroborate the findings of our study.

Other components of the VirTele program that may have also contributed to the participant’s empowerment include the autonomous access to a personalized training program, the motivational aspect of VR exergames (eg, augmented feedback and challenging exercises), shared decision-making using the combination of VR and telerehabilitation, health counseling, and the ability to get real-time feedback during direct or web-based supervision (promoting the desired behavioral result). The extent to which the different VirTele program components played a role in the improvements observed in the clinical outcomes or empowerment indicators is still unclear.

### Study Limitations

First, the FMA-UE results should be carefully interpreted, as the remote administration of the scale without the presence of an evaluator on site with the participant has not been validated in the literature. However, the FMA-UE (motor function) results in this case study were consistent with the self-reported findings (SIS and MAL) and provided an indication of the affected upper extremity motor function pattern during the intervention. The literature lacks a scale that remotely assesses the motor function of the arm in stroke survivors and is extremely relevant to the current need for telerehabilitation interventions.

Second, the participant was already highly motivated when he started the intervention. This may have naturally increased his adherence to the VirTele rehabilitation program and, consequently, favor his clinical outcomes. However, this level of adherence may not be representative of all participants.

In addition, with only 1 participant, it is difficult to capture all the factors that could influence the behavioral intention and use behavior of VirTele, such as technology familiarity, motivation, and previous experiences. The findings gathered so far are limited to the participant’s own experiences, thereby limiting their transferability to other contexts.

The participant was also very comfortable with the technology and easily handled the technical difficulties that occurred during the intervention. This may not represent future users, although the participant’s input during the development phase allowed the team to address potential future technological issues. This is crucial because it is highly likely that many participants affected by a stroke would be older and possibly less comfortable with new information technologies [55].

Finally, we cannot predict how well other participants would manage technical problems or how comfortable they would be with the technology used in the VirTele program. The technical difficulties are considered a direct determinant factor of intervention adherence and may influence a participant’s willingness to use the technology. Technical problems should indeed be considered as a part of technology use, and end users should be technically (where to click and what this signal means) and emotionally (do not panic, you do not need to be frustrated, you did nothing wrong, and that is not your fault) prepared to better manage various situations. Accordingly, a video and informative pamphlet, including simple, concise instructions on how to open and use the exergames and videoconference platform, could be prepared to support VirTele use among future participants.

### Future Research

It is important to explore the extent to which technological knowledge and experience play a role in using the latest technology. In addition, further research is needed to explore the role of shared decision-making and empowerment in exercise program adherence and progression and behavior modification for upper extremity use.

The data collected so far may not be representative of stroke survivors aged 63 years. However, the feasibility and preliminary efficacy results are promising and support the use of the VirTele rehabilitation program and have contributed to improving the intervention and study methodology. Further studies are warranted to corroborate these preliminary findings and test the feasibility and efficacy of VirTele in a diverse stroke population.

### Conclusions

The results of this case study suggest that it is relevant to continue investigating the use of exergames provided by telerehabilitation to improve upper extremity motor function and enhance the upper extremity quality and amount of use in activities of daily living.

## Appendix

Multimedia Appendix 1 Screenshots of the Jintronix exergames.

Multimedia Appendix 2 Guideline for motivational interviewing in the study.

## Abbreviations

BCT: behavior change technique
FMA-UE: Fugl-Meyer Assessment–Upper Extremity
MAL: Motor Activity Log
MCID: minimal clinically important difference
SIS: Stroke Impact Scale
TSRQ: Treatment Self-Regulation Questionnaire
UTAUT: Unified Theory of Acceptance and Use of Technology
VR: virtual reality
