# Supplementary material for: A Personalized Home-Based Rehabilitation Program Using Exergames Combined With a Telerehabilitation App in a Chronic Stroke Survivor: Mixed Methods Case Study
Source: JMIR Serious Games. 2021 Aug 31;9(3):e26153. doi: 10.2196/26153 (PMC8441601; doi:10.2196/26153)
Supplement: Multimedia Appendix 1 [file games_v9i3e26153_app1.pdf]

**Calibration**

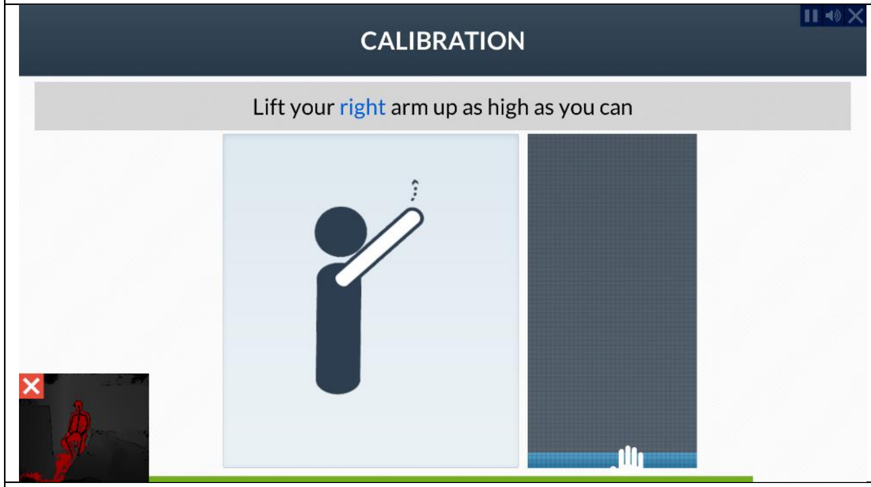


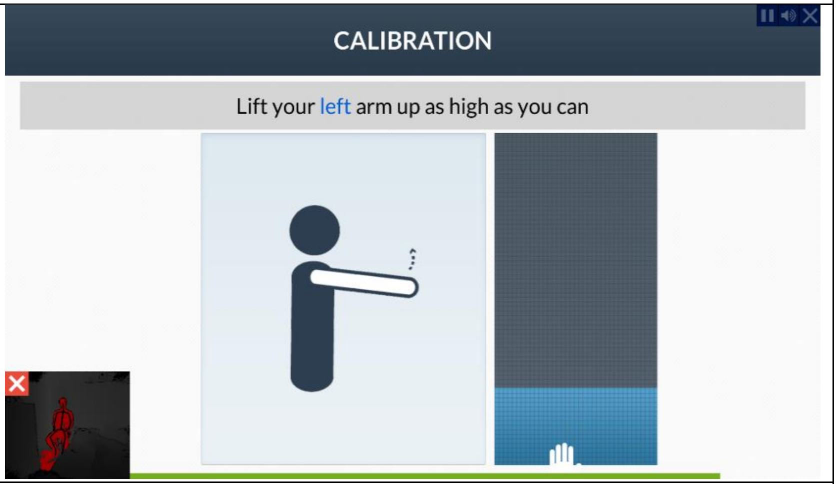


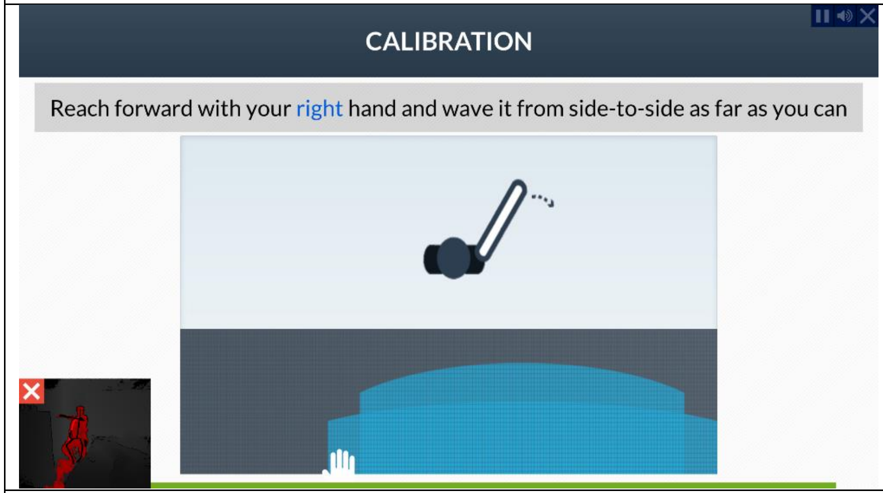


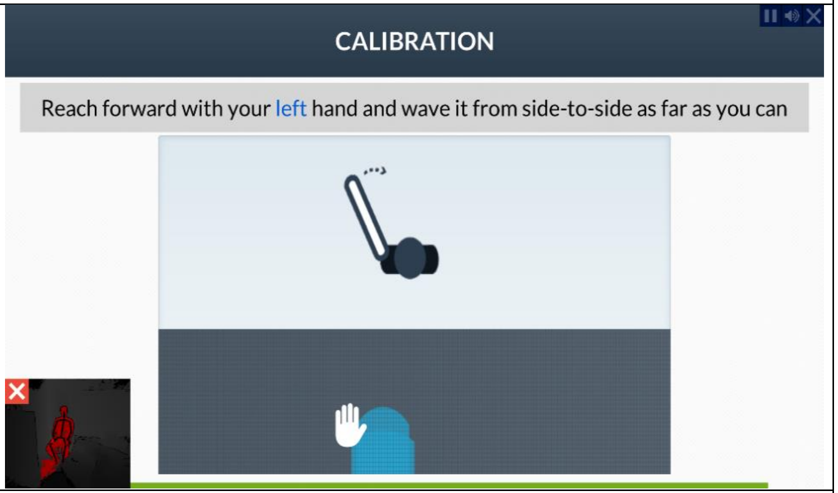


***Multimedia appendix**. Screenshots of Jintronix exergames*

## Space race

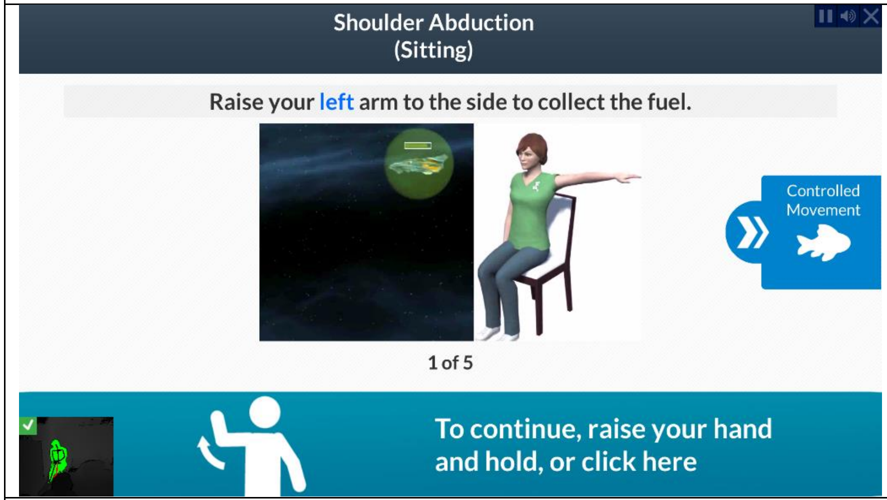


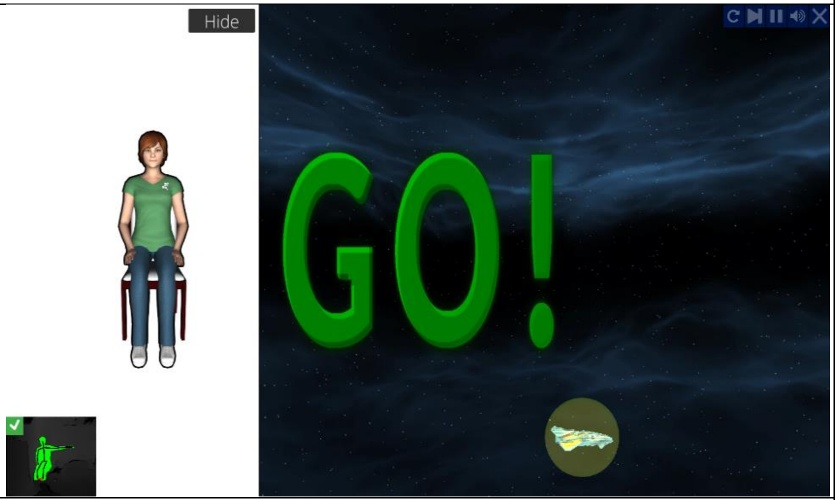


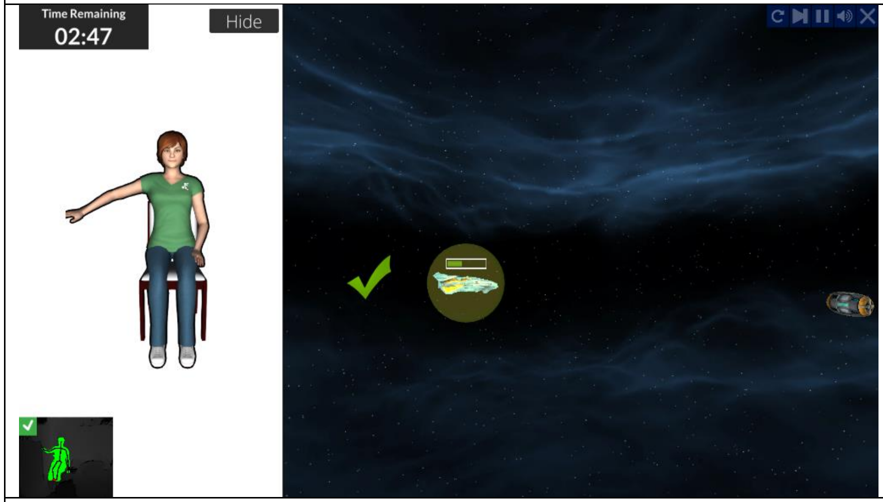


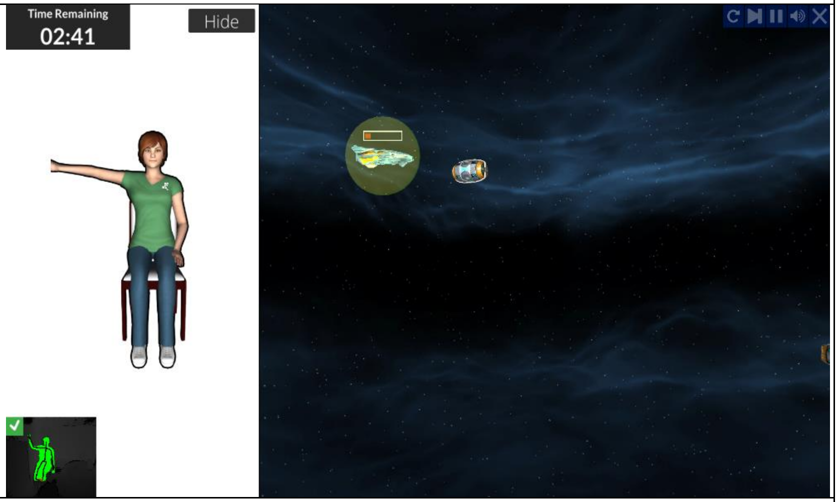


***Multimedia appendix**. Screenshots of Jintronix exergames*

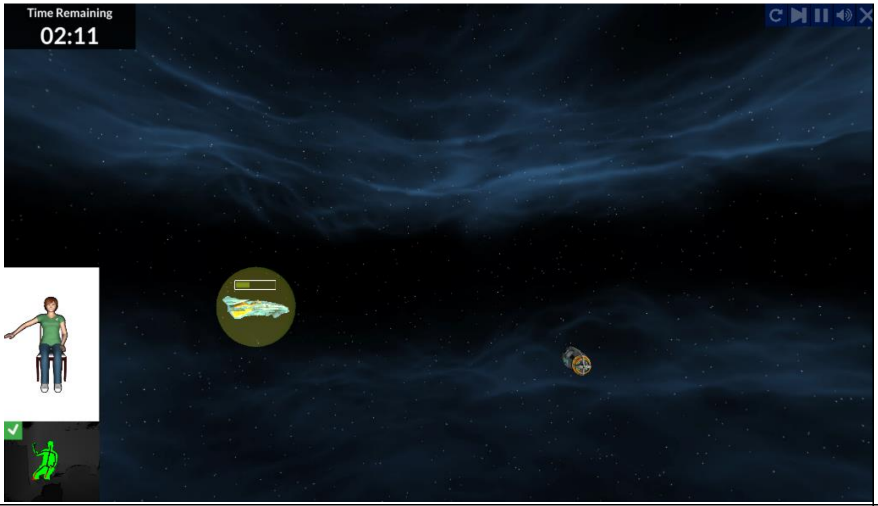


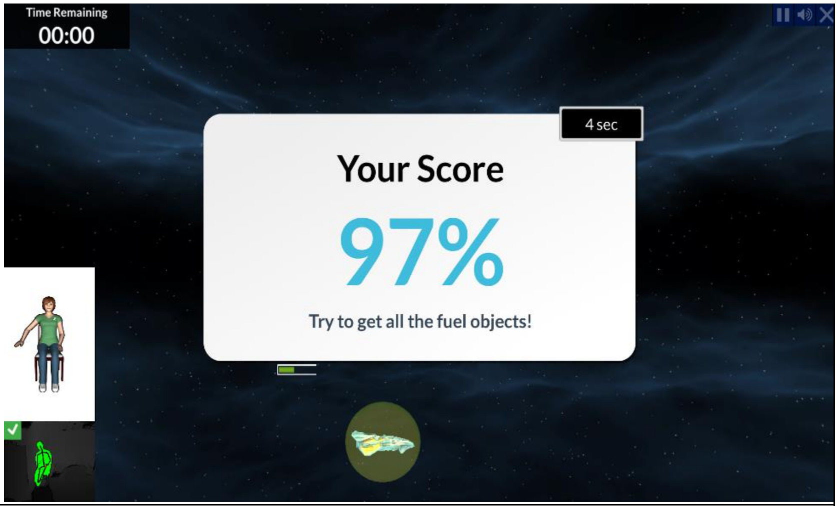


## Catch and Carry an Apple

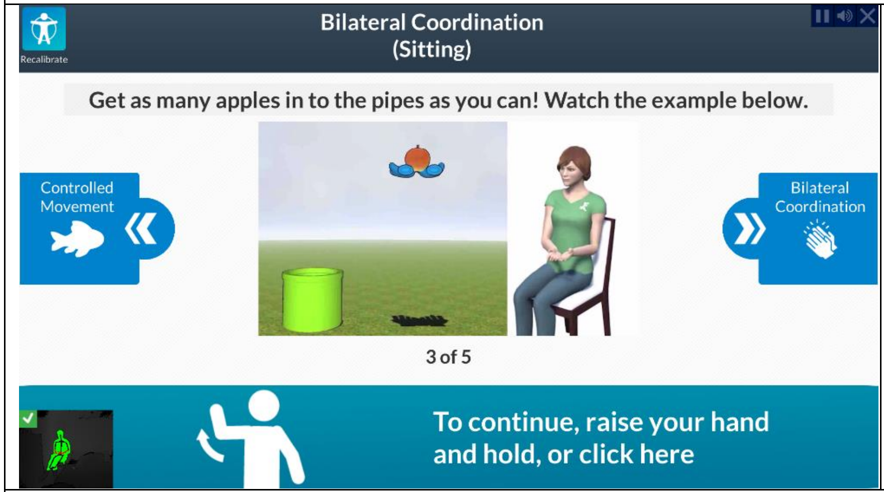


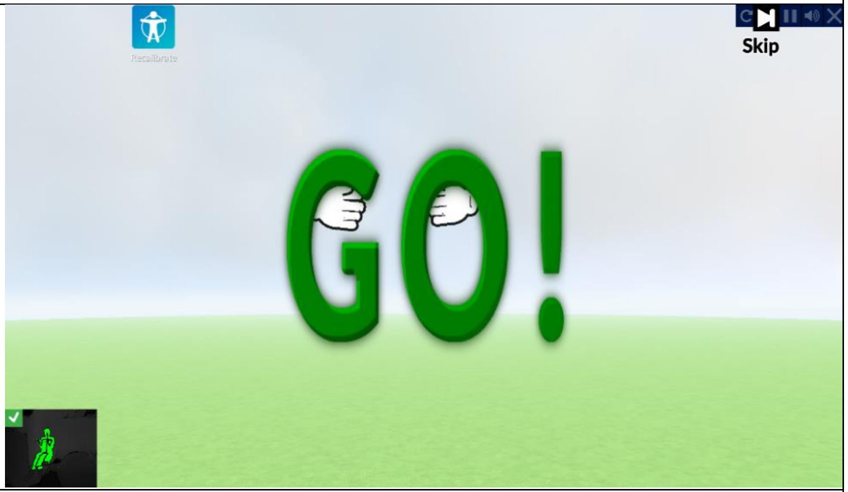


***Multimedia appendix***. *Screenshots of Jintronix exergames*

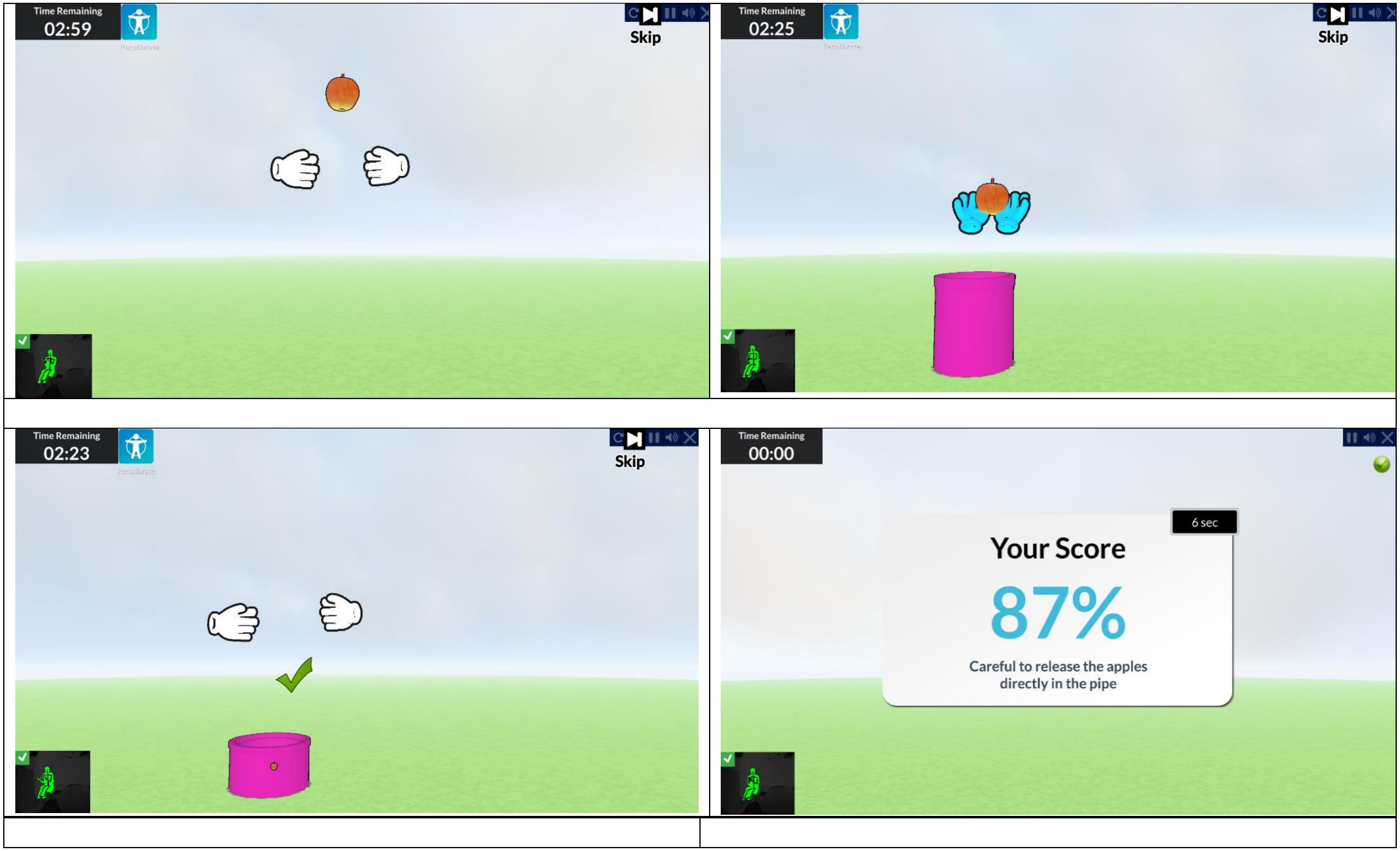


***Multimedia appendix****. Screenshots of Jintronix exergames*

# Pop Clap

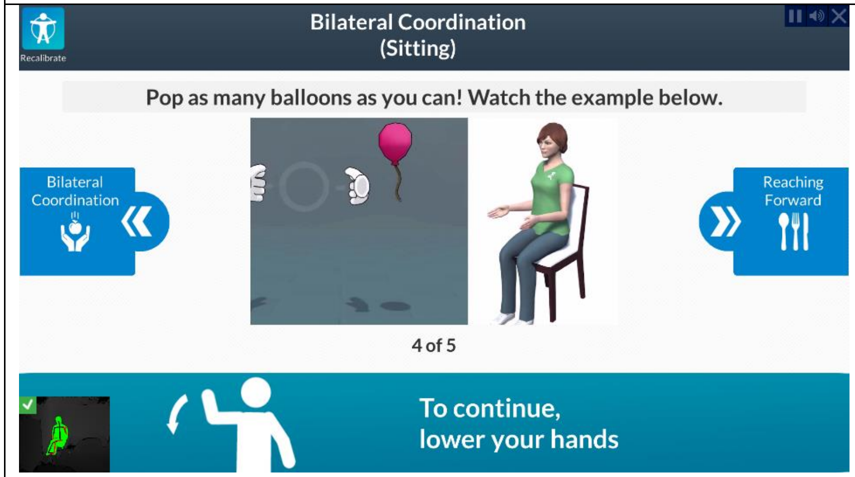


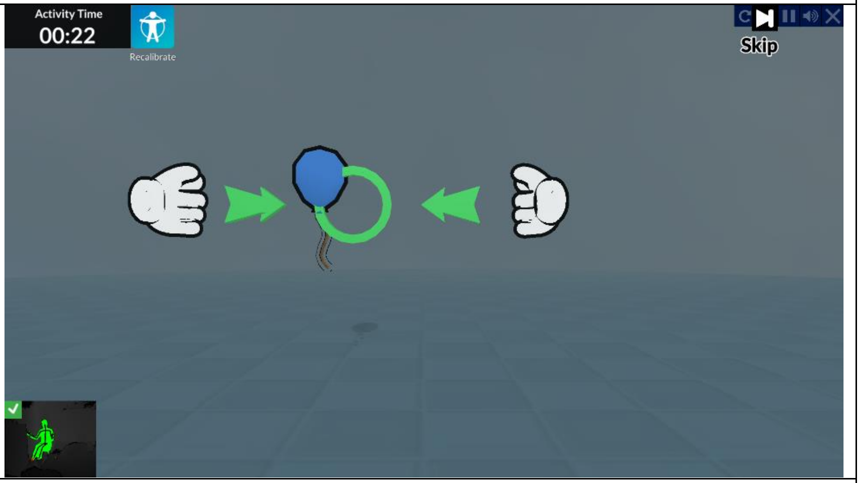


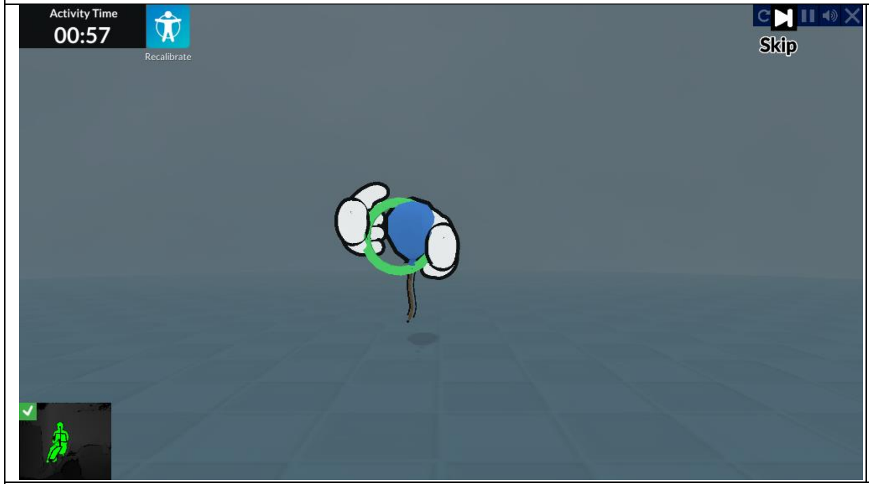


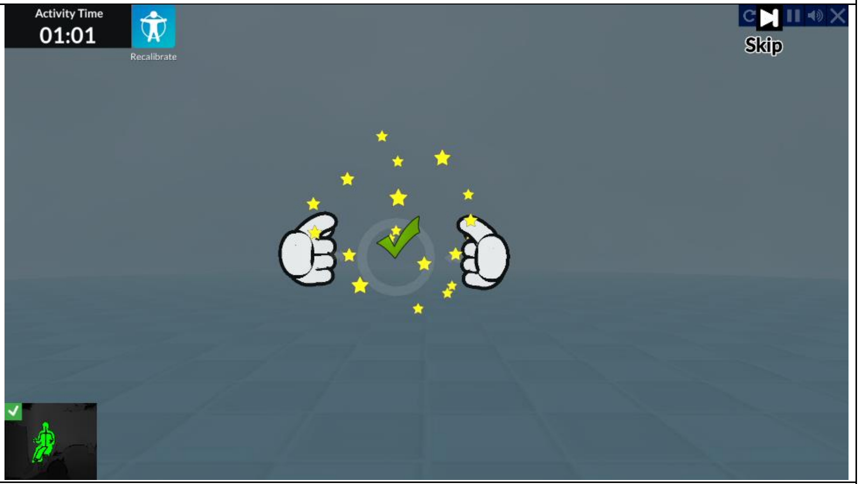


***Multimedia appendix**. Screenshots of Jintronix exergames*

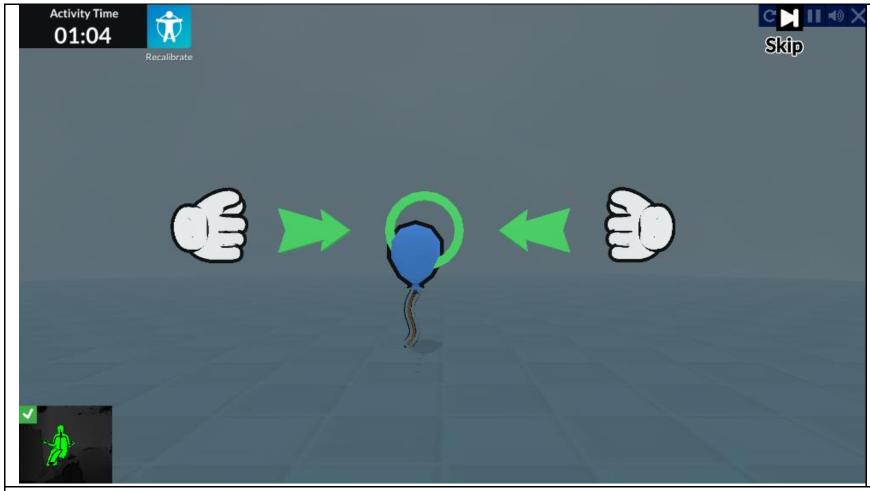


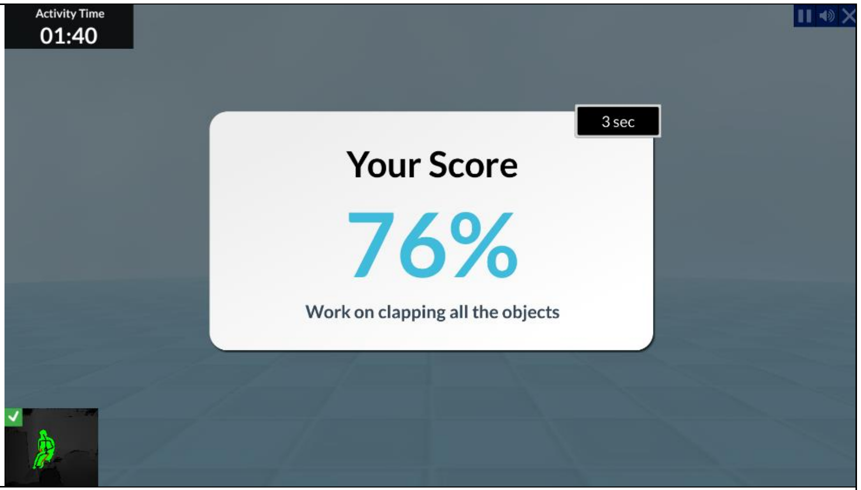


## Kitchen clean up

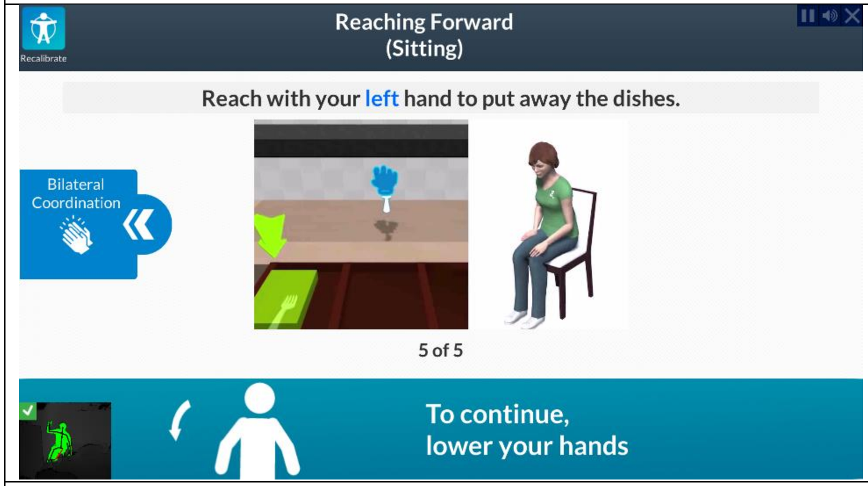


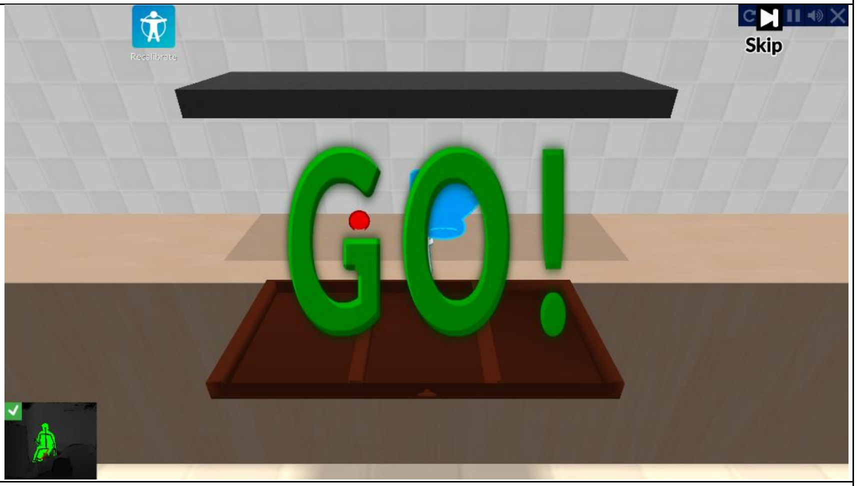


***Multimedia appendix***. *Screenshots of Jintronix exergames*

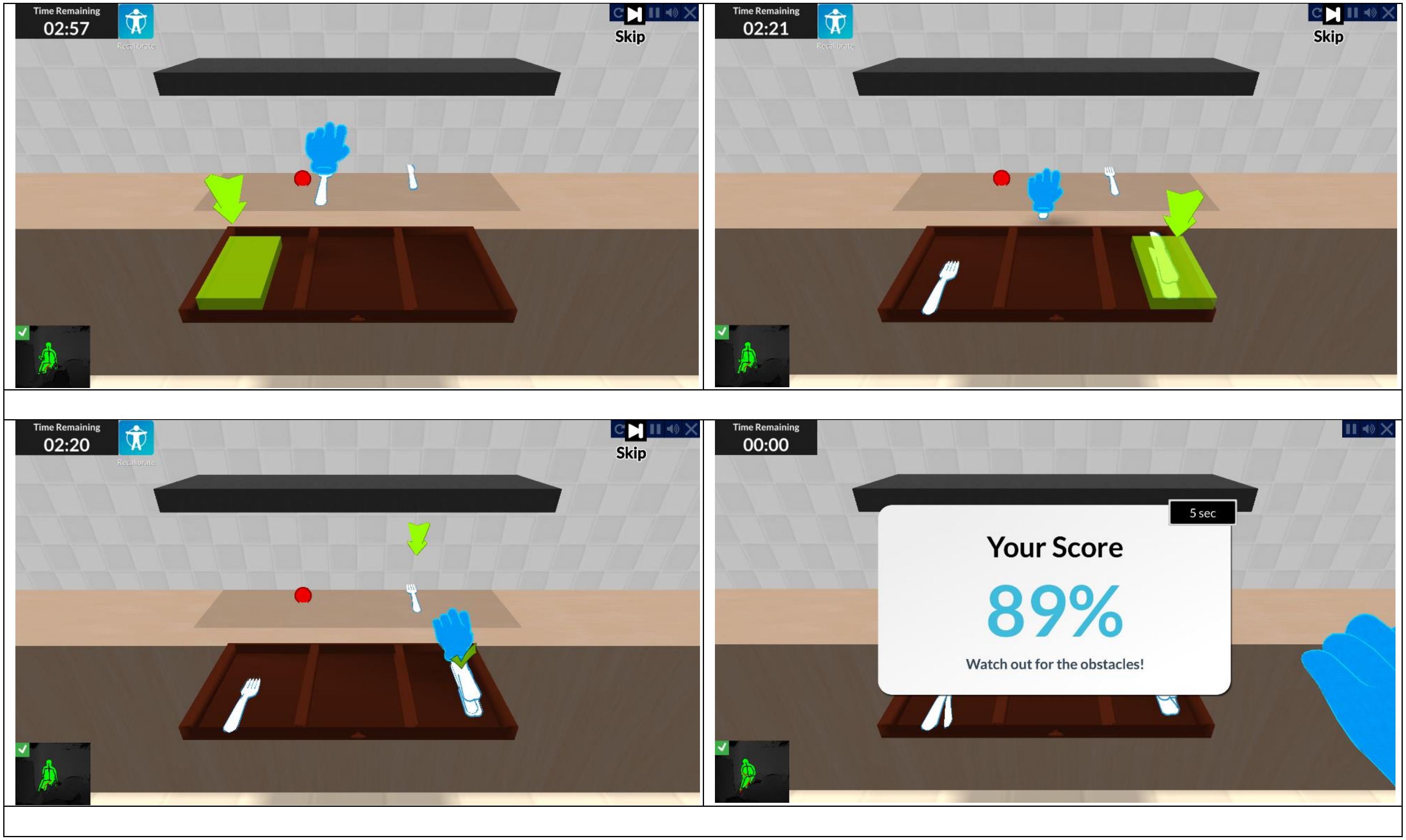


***Multimedia appendix**. Screenshots of Jintronix exergames*
