# Supplementary material for: A Personalized Home-Based Rehabilitation Program Using Exergames Combined With a Telerehabilitation App in a Chronic Stroke Survivor: Mixed Methods Case Study
Source: JMIR Serious Games. 2021 Aug 31;9(3):e26153. doi: 10.2196/26153 (PMC8441601; doi:10.2196/26153)
Supplement: Multimedia Appendix 2 [file games_v9i3e26153_app2.pdf]

Guideline for motivational interviewing in the study

First, the interview begins by asking the participant for permission to discuss his daily use of the affected UE or adherence to exergames (respect autonomy). If the participant uses the affected UE in activities of daily living or adheres to VirTele, the clinician continues with questions related to action planning for maintenance (stimulate competence), for example: Is the pre-identified goal still suitable for you? if not what change do you plan to make to this objective? (1.5 objective revision), How do you plan to organize yourself to use your UE more frequently in the chosen activity (1.4 action planning), What are the possible obstacles to your use of your affected arm and hand during the previously identified activity (s), including relapse and ways to overcome them? (1.2 problem solving).

Second, in the scenario, if the survivor didn't use the affected UE or didn't play exergames at the frequency required, the clinician may continue with questions to explore and resolve ambivalence (while respecting his autonomy), for example, what would be the benefits of keeping things as they are, without changing? (9.2 advantages and disadvantages), If you decide to make a change (eg, to use the affected UE more frequently), what would be some of the challenges you would face? (9.2 advantages and disadvantages). Finally, the clinician is invited to demonstrate empathy towards the participants (connectivity), for example, I am convinced that when you make a firm decision and commit to your exercises, you will find a way to complete them (15.1 verbal persuasion of ability).
